# Do Attentional Lapses Account for the Worst Performance Rule?

**DOI:** 10.3390/jintelligence10010002

**Published:** 2021-12-24

**Authors:** Christoph Löffler, Gidon T. Frischkorn, Jan Rummel, Dirk Hagemann, Anna-Lena Schubert

**Affiliations:** 1 Institute of Psychology, Heidelberg University, Hauptstr. 47-51, 66917 Heidelberg, Germany; christoph.loeffler@psychologie.uni-heidelberg.de (C.L.); jan.rummel@psychologie.uni-heidelberg.de (J.R.); dirk.hagemann@psychologie.uni-heidelberg.de (D.H.); 2 Department of Psychology, University of Mainz, Wallstr. 3, 55122 Mainz, Germany; 3 Department of Psychology, University of Zurich, Binzmühlestr. 14, 8050 Zurich, Switzerland; gidon.frischkorn@psychologie.uzh.ch

**Keywords:** worst performance rule, attentional lapses, attentional lapses account, intelligence, multilevel analysis, task-unrelated thoughts

## Abstract

The worst performance rule (WPR) describes the phenomenon that individuals’ slowest responses in a task are often more predictive of their intelligence than their fastest or average responses. To explain this phenomenon, it was previously suggested that occasional lapses of attention during task completion might be associated with particularly slow reaction times. Because less intelligent individuals should experience lapses of attention more frequently, reaction time distribution should be more heavily skewed for them than for more intelligent people. Consequently, the correlation between intelligence and reaction times should increase from the lowest to the highest quantile of the response time distribution. This attentional lapses account has some intuitive appeal, but has not yet been tested empirically. Using a hierarchical modeling approach, we investigated whether the WPR pattern would disappear when including different behavioral, self-report, and neural measurements of attentional lapses as predictors. In a sample of *N* = 85, we found that attentional lapses accounted for the WPR, but effect sizes of single covariates were mostly small to very small. We replicated these results in a reanalysis of a much larger previously published data set. Our findings render empirical support to the attentional lapses account of the WPR.

## 1. Introduction 

**Figure 1:**
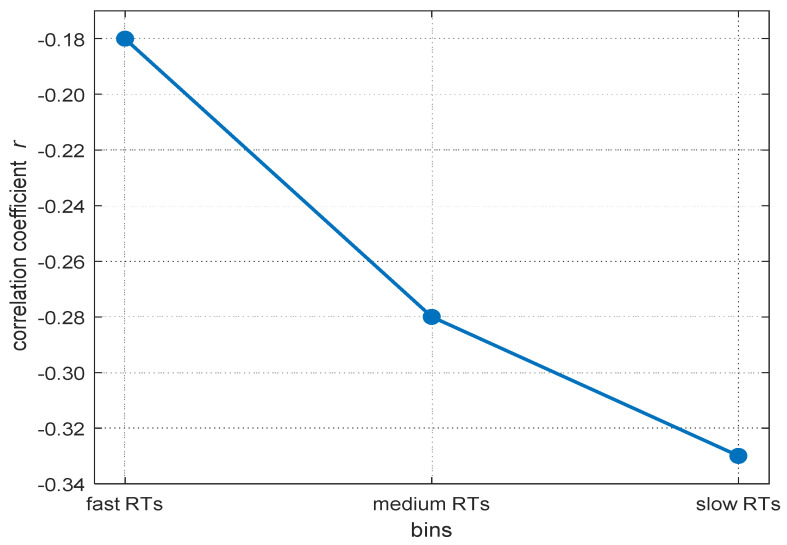
An example for the increasing magnitude in correlations between RT and mental abilities from fast to slow RT-bins. Data are based on the meta-analysis from Schubert (2019).

Reaction times (RTs) in elementary cognitive tasks typically correlate moderately with general intelligence (Doebler and Scheffler 2016; Sheppard and Vernon 2008). Moreover, if intra-individual RT distributions are divided into bins from the fastest to the slowest RTs, the negative relations between mean RT within each bin and intelligence increase from the fastest to the slowest parts of the distribution (Baumeister and Kellas 1968; Coyle 2003; Larson and Alderton 1990; Schubert 2019). Larson and Alderton (1990) named this phenomenon the worst performance rule (WPR). The WPR suggests that inter-individual differences in slower RTs explain more of the variance in individuals’ cognitive abilities than faster RTs (see Figure 1 for an illustration of the typical WPR pattern). As pointed out by Larson and Alderton (1990), a better understanding of this phenomenon is desirable as it may be informative of the cognitive mechanisms underlying inter-individual differences in intelligence. 

The WPR has been observed in several studies (Diascro and Brody 1993; Fernandez et al. 2014; Frischkorn et al. 2016; Kranzler 1992; Leite 2009; McVay and Kane 2012; Rammsayer and Troche 2016; Schmiedek et al. 2007; Schmitz et al. 2018; Schmitz and Wilhelm 2016; Unsworth et al. 2010), although there are a few studies that did not find evidence for a WPR (Dutilh et al. 2017; Ratcliff et al. 2010; Salthouse 1993, 1998; Saville et al. 2016). A recent meta-analysis addressed the question of the strength, consistency, and generalizability of WPR across 23 datasets (from 19 different studies and 3767 participants) and found evidence in favor of the WPR (Schubert 2019). 

Identifying the underlying mechanisms of the WPR may help to identify the elementary processes producing inter-individual differences in intelligence, because whichever process is measured particularly with the slowest RTs may also contribute to differences in mental abilities. Different candidate accounts for explaining the occurrence of the WPR have been proposed. Several authors suggested an attentional lapses account of the WPR which states that the WPR occurs due to lapses of attention to which less intelligent people are particularly prone (Jensen 1992; Larson and Alderton 1990; Unsworth et al. 2010). On a neural level, this could be reflected by less intelligent individuals showing a higher frequency of neural transmission errors (Coyle 2001; Miller 1994) or spending more processing time on neural subthreshold and refractory periods, resulting in errors or delays during information processing (Jensen 1992). As the attentional lapses account is currently the most prominent account for explaining the WPR, we put this account at critical test in the present study. 

### 1.1. The Attentional Lapses Account of the WPR and Its Examination

According to the executive attention theory of working memory (Kane et al. 2008), individual differences in executive attention predict differences in working memory capacity (WMC) and higher cognitive abilities such as fluid intelligence. While performing any type of (demanding) cognitive tasks, external distractors (such as a loud noise) and internal distractors (such as thoughts about the last or next vacation) may interfere with task completion by impairing task processing and goal maintenance. Accordingly, individuals who are able to shield their current thoughts against such task-irrelevant external or internal distractors should show better task performance. Kane et al. (2008) suggested that certain people are better at blocking out task-irrelevant information and maintaining current task goals than others, in particular those people with high executive attention (see also Kane et al. 2004). Individuals with lower executive attentional control, however, should perform worse in cognitive ability tests, because they are not able to keep their attention adequately focused on a task.

The consequence of such executive attention failures is that people who are not able to focus their attention on the task at hand experience attentional lapses while performing a task. Empirically, this will result in slower correct responses or in fast response failures (Unsworth et al. 2010). From an individual differences perspective, one would therefore expect that individuals with a higher propensity for attentional lapses occasionally show very slow but correct responses or a higher error rate. In fact, previous research has shown that self-reported attentional lapses were moderately associated with individual differences in the right tail of the RT distribution, that is, individuals who reported higher rates of attentional lapses showed more positively skewed RT distributions and hence more frequent slow responses (McVay and Kane 2012). In addition, self-reported attentional lapses predicted error rates in simple experimental tasks such as the sustained attention to response task (McVay and Kane 2009; Smallwood and Schooler 2006). 

If individual differences in attentional lapses are related to differences in cognitive abilities such as fluid intelligence and if attentional lapses lead to slow responses, it is in consequence not surprising that slower responses are more strongly related to intelligence than fast responses (i.e., the phenomenon of the WPR). In contrast to faster responses, slower RTs reflect attentional lapses as an additional process, which results in the typical pattern of the WPR. Additional analyses by McVay and Kane (2012), in which they demonstrated that individual differences in self-reported attentional lapses partly mediated the association between slowest RTs and WMC, provided first evidence supporting this hypothesis.

### 1.2. Multiverse Manifestation and Measurement of Attentional Lapses

Attentional lapses are a multi-faceted construct (Robison et al. 2020) and that is the reason why the measurement of attentional lapses is not straightforward. There are different possibilities to operationalize participants’ attentional states (McVay and Kane 2012; Unsworth et al. 2010). Most of the measurements—which we used in this study—were adapted from mind wandering research and possess face validity to the construct of attentional lapses. Possible manifestations of attentional lapses can be found in participants’ self-reported attentional states, their response behavior, or psychophysiological measures. 

Many studies measured attentional lapses as participants’ self-reported mental states (Smallwood and Schooler 2015). During an ongoing task, participants are typically asked whether their thoughts are on- or off-task. In consequence, if their thoughts are not on the ongoing task, they are experiencing task-unrelated-thoughts (TUTs; Smallwood and Schooler 2006), which are considered as attentional drifts or attentional lapses (McVay and Kane 2010; Watkins 2008). Individuals tend to show a larger variability in those RTs in which they report TUTs, but they do not consistently show shifts of mean RTs (Leszczynski et al. 2017; McVay and Kane 2009, 2012; Seli et al. 2013, 2014; Thomson et al. 2014). These results suggest that attentional lapses may lead to an increase in the variability of RTs due to occasional failures in an experimental task.

Another method to measure the subjective frequency of attentional lapses are questionnaires that measure participants’ attentional states during everyday life experiences and their personal tendencies for attentional lapses in everyday situations. Individuals who reported a higher tendency for attentional lapses also tended to report a higher frequency of TUTs during experimental tasks (Mrazek et al. 2013; Schubert et al. 2020). This suggests that both measurements assess—at least to some degree—the same underlying construct. 

As a more objective alternative, it has been proposed to assess attentional states with behavioral measures such as the metronome response task (MRT; Seli et al. 2013). This task measures attentional lapses based on intraindividual variability in participants’ tapping response to a continuous metronome beat. It has been suggested that individuals’ tapping variance may reflect their attentional states (Seli et al. 2013). Seli et al. (2013, 2014) showed that self-reported attentional lapses are related to increases in tapping variability on the metronome beat in this task.

Beyond behavioral and self-report measures, former research identified several electrophysiological correlates of attentional lapses. The P3 is a component of the event-related potential (ERP) that occurs about 300 ms after stimulus onset at parietal electrodes and is associated with a wide range of higher-order cognitive processes such as stimulus evaluation and memory updating (Polich 2007; Verleger 2020). It has been repeatedly associated with self-reported attentional lapses: Several studies found reduced P3 amplitudes during trials in which participants reported not having been focused on the task (Kam and Handy 2013; Smallwood et al. 2008). The same pattern of results was reported by Barron et al. (2011), who found a reduced P3 amplitude in participants who experienced more attentional lapses in comparison to more focused participants. In addition, attentional lapses have been shown to affect sensory processing, as smaller visual P1 amplitudes have been observed for trials in which participants reported attentional lapses (Baird et al. 2014; Kam et al. 2011; see also Kam and Handy 2013). The P1 is a component of the event-related potential that occurs about 100 ms after stimulus onset at occipital electrodes. These findings suggest that attentional lapses affect the neurocognitive processing of information and that they are accompanied by a reduction of amplitudes of ERP components associated with stimulus perception and evaluation.

Furthermore, several studies reported that attentional lapses were associated with changes in the time-frequency domain, in particular with increases in inter-stimulus alpha power and increases in stimulus-locked theta power. Alpha activity is known to reflect an internally oriented mental state (Hanslmayr et al. 2011) and has, for example, been shown to increase during episodes of mental imaging (Cooper et al. 2003) and to be suppressed during sensory stimulation (Berger 1929; Thut et al. 2006). Episodes during which attention is not fully oriented towards the actual task have therefore been associated with greater alpha power (Baldwin et al. 2017; Compton et al. 2019; O’Connell et al. 2009). Arnau et al. (2020) further disentangled the time-course of this association and found alpha power to increase overall, but particularly at lateral parietal and occipital electrodes during the inter-trial-interval before participants reported TUTs. This internal focus of attention was redirected to the primary experimental task once an imperative stimulus (e.g., the fixation cross) was presented.

Theta power, especially event-related frontal-midline theta power, is associated with executive control and regulation processes (Cavanagh et al. 2012; Cavanagh and Frank 2014). Previous research has suggested that theta power may decrease when attentional lapses occur and may be subsequently upregulated as a compensatory mechanism once attentional drifts are noticed (Arnau et al. 2020; Atchley et al. 2017; Braboszcz and Delorme 2011). This redirection of attention towards the primary task may be initiated by either meta-awareness regarding one’s attentional state (Braboszcz and Delorme 2011; Smallwood et al. 2007) or by external cues such as the presentation of the fixation cross or the next experimental trial (Arnau et al. 2020). 

To achieve a most comprehensive analysis in the present study, we combined these heterogeneous approaches and applied a multiverse strategy for capturing participants’ attentional states with different operationalizations in a multimethod approach. Therefore, we combined the listed self-report measurements with the listed behavioral and psychophysiological measures.

### 1.3. Identifying Occurrences of the WPR

In the present study, we analyzed the WPR before and after controlling for individual differences in attentional lapses by applying a recently proposed multilevel approach to the WPR (Frischkorn et al. 2016). Most WPR studies reported only the correlations of the mean or median RTs in the performance bands with intelligence, which is merely a description of the WPR rather than an inferential statistical examination of the phenomenon. If studies tested increasing correlations over RT bands for significance, they used rank-correlations (e.g., Kranzler 1992; Larson and Alderton 1990) or comparisons of correlation coefficients from dependent samples with Fisher’s *Z*-values (e.g., Rammsayer and Troche 2016). Both statistical methods have certain weaknesses. 

One weakness of rank-correlations is that they only quantify the extent of monotonicity in the increase of negative covariances or correlations between RTs and intelligence over the different bins. If this increase is quite monotonic, a rank-correlation close to one will be found no matter how large the increase is. By using the rank-correlation as a method to test the WPR, it is not possible to quantify the slope of the increase of correlations over bins of the RT distribution, which is needed to quantify the size of the WPR. The second weakness of rank-correlations is that they ignore the estimation uncertainty of correlations if these correlations are first estimated and then subsequently entered as observed variables into rank-correlations. This sequential approach results in a possible overestimation of the significance of the WPR (Skrondal and Laake 2001). Moreover, tests assessing the difference between dependent correlations suffer from low statistical power, possibly underestimating the WPR. For these reasons, we used the recently proposed multilevel account to test the WPR more adequately, i.e., in a single estimation step and with higher statistical power (Frischkorn et al. 2016).

There are two possible ways to measure the worst performance pattern by using either unstandardized (covariances) or standardized (correlations) coefficients in the multi-level models. On the one hand, covariances reflect the unstandardized relation between two variables, which means that an increase in magnitude of covariances can have two reasons: They can either reflect an actual increase of the relation between both variables or they can reflect increases in inter-individual variances in at least one of the two variables. On the other hand, increasing correlations represent increases in the relationships between two variables, because correlations are controlled for inter-individual variances. To understand attentional lapses’ influences on the RT variances and their effects on the relation between RT and intelligence, we used both unstandardized and standardized coefficients in the present analyses. In order to obtain a higher resolution of the course of the WPR and the influence of attentional lapses on the WPR, we analyzed the RT distribution on trial-by-trial basis with multilevel models and did not apply a binning procedure as, e.g., Frischkorn et al. (2016) did.

The aim of the present study was to assess if individual differences in the frequency of attentional lapses could account for the WPR. Due to the multiverse nature of attentional lapses, we used behavioral, self-report, and electrophysiological methods to assess individual differences in the frequency of attentional lapses. In addition, we used the previously proposed multilevel account of the WPR (Frischkorn et al. 2016) to quantify and test any moderating effect of attentional lapses on the strength of the worst performance effect. Based on the attentional lapses account, we assumed that individual differences in attentional lapses explain—at least partially—the emergence of the WPR. Hence, we expected the slope of the WPR to be significantly reduced if we controlled RTs for individual differences in attentional lapses.

## 2. Study 1

### 2.1. Materials and Methods

The study was approved by the ethics committee of the faculty of behavioral and cultural studies of Heidelberg University. At the beginning of an experimental session, participants signed an informed consent.

#### 2.1.1. Participants

We recruited a sample of *N* = 100 general population participants via the local newspaper, via our own university homepage, via a pool of potentially interested participants in psychological studies, and by distributing flyers in Heidelberg. All volunteers were admitted if they were between 18 and 60 years old and had no history of mental illnesses. Two participants were removed because they did not complete the experiment. In consequence of the outlier analysis (see below), 13 more participants were removed from further analyses. The remaining sample (*N* = 85) consists of 29 males and 56 females. Participants’ mean age was 30.21 years (*SD* = 12.33). All participants either stated that German was their mother tongue or that they spoke German on a level comparable to native speakers. The educational degrees were distributed in the following way: As highest educational level, 49 participants had a high school diploma (German Abitur), 30 had a university degree, and six had an educational degree lower than a high school diploma. All participants had normal or corrected to normal vision. They received 30 € and personal feedback as compensation for their participation.

#### 2.1.2. Materials

##### Berlin Intelligence Structure Test (BIS)

To measure participants’ intelligence, we used the short version of the Berlin Intelligence Structure Test (BIS-4, booklet 2: Jäger et al. 1997). The short version of the BIS is a particularly suitable instrument for measuring cognitive abilities in a relatively short time (about 50–60 min). Moreover, the short version of the BIS is a heterogeneous test battery for different abilities and includes 15 different tasks. Based on the theory by Jäger (1984), the test consists of four operation-related (processing speed, memory, creativity, processing capacity) and three context-related (verbal, numerical, figural) components of intelligence. Furthermore, the test allows the calculation of a general intelligence (*g*) score. We used the sum scores across all subtests as an independent variable. 

Five participants had already completed the same test within the last year at our department. Because there may be a training effect between the two measurement occasions within one year (Scharfen et al. 2018), we used their BIS-scores from the previous study for all further analyses. The mean test score of the whole sample (*N* = 85) was 1498.29 (*SD* = 80.02) which corresponds to a converted mean IQ score of 94.58 (*SD* = 16.12). Cronbach’s α showed a good internal consistency for the test scores (α = .79).

##### Choice RT Task: Switching Task

We measured RTs in a switching task, which was based on a task used by Sudevan and Taylor (1987). An unpublished reanalysis of a previous study in which we used this task (Frischkorn et al. 2019) suggested that it yields a significant worst performance effect.

While participants were working on this task, they had to decide whether a presented digit was smaller or larger than five or whether it was an odd or an even number. This task is constructed based on a 2 × 2 design and consists of four different experimental conditions. Which rule currently applied depended on the color in which the stimuli were presented (red = less/more condition, green = odd/even condition). The digit of a single trial could be either presented in the color of the former trial (=repeat condition) or in the other color (=shifting condition). The stimulus set included the digits between one and nine, excluding five. 

The task was programmed in MATLAB (The MathWorks Inc., Natick, MA, USA) with the open source software package Psychtoolbox version 3.0.13 (Kleiner et al. 2007). We implemented restrictions that the same digits could never appear twice in a row as well as the same color could never appear more than three times in a row. Participants were instructed to answer as correctly and as fast as possible. On the keyboard, they had to press “L” to indicate that a digit was either larger than five or even and they had to press “D” to indicate that a digit was either smaller than five or odd.

**Figure 2:**
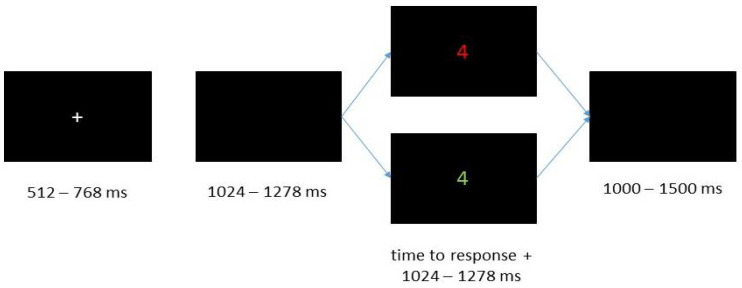
Representation of the sequence of one trial.

All stimuli were presented in the middle of the screen on a black background (Figure 2). At the beginning of each trial, a gray fixation cross was shown for 512–768 ms. Following the fixation cross, a blank screen was presented as inter stimulus interval for 1024–1278 ms. Subsequently the digit followed and disappeared 1024–1278 ms after the participants responded. The stimulus disappeared after three seconds if the participants did not respond. At the end of each trial a blank screen was presented again as an inter-trial interval of 1000–1500 ms.

Participants completed 40 practice trials (ten trials task pure less/more, ten trials task pure odd/even, and 20 trials including task shifting) during which they received feedback. After that, they worked on the experimental trials, which consisted of ten blocks with 64 trials each. Participants took self-paced breaks between the blocks.

##### Online Thought-Probing Procedure

We administered an online thought-probing procedure by monitoring TUTs with a binary either/or question (see Weinstein 2018). This method is a subjective self-report in which the participants are intermittently asked what their current state of mind is (on task/off task) while they are working on a task. This report is one of the most frequently used methods for capturing online mind wandering at the moment of occurrence (Weinstein 2018).

Participants were randomly asked about TUTs between every fifth and tenth trial. The question was: “Where have you been with your thoughts right now?” Participants could either answer “on task” or “not on task” by pressing the right or left arrow key on the keyboard. On average, participants were probed 91.62 times (*SD* = 2.16) for TUTs while they worked completed 640 trials of the experimental task. On-task-reports were coded as 0 and off task reports were coded as 1 in our data. 

##### Questionnaire of Spontaneous Mind Wandering (Q-SMW)

We used a nine-items measure of spontaneous mind wandering to assess trait mind wandering. For this we combined five items of the Mind Wandering Questionnaire (MWQ; Mrazek et al. 2013) and four items of a scale measuring spontaneous mind wandering (Carriere et al. 2013) into one questionnaire. Participants could answer these questions on a seven-point Likert scale from “almost never” (coded as 1) to “almost always” (coded as 7). Cronbach’s α showed a good internal consistency (α = .81). Because the original items were in English, they were translated into German by two people and translated back into English by another person. We present two items as examples to show their original wording and their context: “I have difficulty maintaining focus on simple or repetitive work” (Mrazek et al. 2013); “I find my thoughts wandering spontaneously” (Carriere et al. 2013).

##### Metronome Response Task (MRT)

We used the MRT as a more objective behavioral assessment of attentional lapses. This task was developed by Seli et al. (2013) as a new method measuring mind wandering that does not rely on self-reports. In the MRT, participants had to answer to the rhythmic beat of a metronome. A larger variability in responses (measured as the standard deviation of discrepancy) is supposed to indicate a higher frequency of attentional lapses, as lapses in executive control are thought to increase behavioral variability.

Participants heard a rhythmic metronome beat every 1600 ms for 400 times while they were looking at a black screen. They were instructed to press the spacebar on the keyboard simultaneously to the sound/rhythmic beat. We calculated the standard deviation of participants’ response discrepancy from the metronome beat after discarding the first five trials as a measure of attentional lapses.

##### Electrophysiological Correlates of Attentional Lapses

The EEG was recorded during the switching task. Based on previous findings, we chose mean amplitudes of lateral occipital P1 (time window: 100–140 ms after stimulus onset), central parietal P3 (time window: 300–630 ms after stimulus onset), pre-fixation cross parieto-occipital alpha power (from 1000 to 200 ms before the onset of the imperative fixation cross) from central and dorsolateral electrodes, and post fixation cross fronto-central theta power (from 0 to 500 ms after the onset of the imperative fixation) as electrophysiological covariates representing attentional lapses.

#### 2.1.3. Procedure

After participants signed an informed consent, they completed the intelligence test under the supervision of the experimenter. This took between 50 and 60 min. After that, electrodes were administered to the scalp and participants were seated in a sound-attenuated, dimly lit cabin. Subsequently, participants worked on the switching task, working memory tasks (not included in the present manuscript), and the MRT in the same order. At the end of the session, participants completed the Q-SMW as well as a questionnaire for the assessment of demographic data. The whole procedure lasted about 3.5 h.

#### 2.1.4. EEG Recording

While participants worked on the switching task the EEG was recorded with 32 equidistant Ag/AgCl electrodes (32Ch-EasyCap, EASYCAP, Herrsching, Germany) and amplified by a BrainAmp DC amplifier (Brain Products, Gilching, Germany). For more information on electrode positions, see Figure S1 in the Supplementary Materials. We used the aFz electrode as the ground electrode. All electrodes were initially referenced to Cz and offline re-referenced to an average reference. For the whole time we kept impedances of all electrodes below 5 kΩ. The EEG signal was recorded continuously with a sampling rate of 1024 Hz (high-pass 0.1 Hz).

#### 2.1.5. Data Analyses

For data preparation and analyses we used the statistics software R—version 4.0.0 (R Core Team 2021). The following packages were used in R: For data processing and easier data management the package “tidyverse”(Wickham et al. 2019), for estimating Cronbach’s α the package “psych” (Revelle 2020), for estimating multilevel models the package “lme4” (Bates et al. 2015) and the “optimx” algorithm (Nash and Varadhan 2021), for estimating the degrees of freedom in the multilevel models the package “lmerTest” (Kuznetsova et al. 2017), and for estimating the effect-sizes the package “effectsize” (Ben-Shachar et al. 2020). For preprocessing and quantification of EEG measures, we used EEGLAB (Delorme and Makeig 2004) and ERPLAB (Lopez-Calderon and Luck 2014) open source toolboxes on MATLAB 2018a (The MathWorks Inc., Natick, MA, USA).

##### Analysis of Behavioral and Self-Report Data

Responses faster than 150 ms and incorrect responses were discarded. Furthermore, the two trials following an online thought probe were excluded from the dataset, because thought probes may interrupt the ongoing task (Steindorf and Rummel 2020). Next, we conducted an intraindividual outlier analysis of the remaining trials and discarded all trials with RTs that deviated more than 3 *SD*s from the mean of the intraindividual logarithmic RT distribution. We conducted a careful outlier analysis, because outlier trials should not have any influence on the occurrence of the WPR (Coyle 2003).

In addition, participants with extremely low (sum score ≤ 1316) or high (sum score ≥ 1747) BIS performance were removed from further analyses. These cut-off values correspond to *z*-values <−3 and >3, which would be considered as clear outliers. This led to the exclusion of five datasets from further analyses. Moreover, we removed one additional participant because they had a mean RT that deviated more than 3 *SD*s from the sample mean.

**Figure 3:**
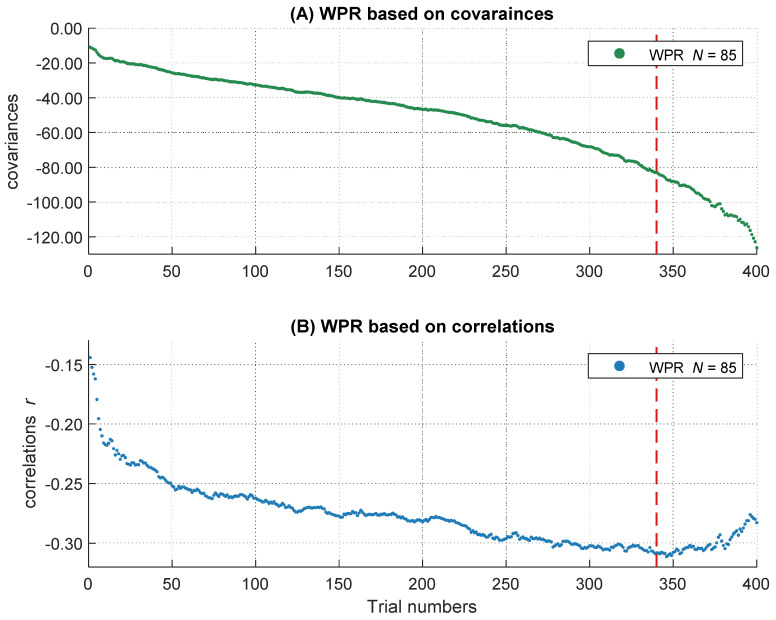
The increasing magnitude of negative correlations and covariances over RT distribution. The course of the covariances over 400 trials is shown above (**A**), the course of correlations over 400 trials is shown below (**B**). The dashed line represents the 85 percent threshold. Only the left part of the red dashed line was analyzed in the following multi-level analyses.

To get the full information of the whole RT distribution, we decided not to summarize individual RTs in several bins, but to use information of every trial within each participant. Therefore, after the outlier analyses, we sorted all remaining trials in ascending order according to their RTs. All participants with at least 400 correct responses were included to ensure a sufficient and comparable number of trials across participants on the one hand and to minimize the number of participants with fewer trials who had to be excluded from the analyses on the other hand. This led to a final sample of 85 participants. We used the middle 400 trials of each participant’s RT distribution and removed the remaining trials symmetrically from both ends of each intraindividual RT distribution. Single trial RTs served as the dependent variable in the following analyses. However, in the slowest 15 percent of the trials, the increases in the magnitude of the covariances accelerated whereas the negative relations became smaller (see Figure 3 and also General Discussion). As this course does not correspond to the definition of the WPR, which assumes a monotonic increase of correlations, we analyzed only the fastest 85 percent of the trials (340 trials). Further, we centered the data to the middle trial of each participant’s RT distribution and rescaled the trial numbers in the range from −2 to 2. The central trial with the rescaled value 0 is equivalent to the trial with the number 170 and the trials with the values −2 and 2 correspond to the fastest trial 1 and the slowest trial 340. This is important for the interpretation of the *b*-weights in the multilevel models, both for the main effects and the interaction terms.

##### Preprocessing of Electrophysiological Data for Event-Related Potentials (ERPs)

Only correct trials were included. EEG data were filtered with an offline band-pass filter of 0.1–30 Hz. Bad channels were identified based on probability, kurtosis, and spectrum of the channel data. Data were down sampled to 512 Hz. Then, the stream of EEG data was divided into epochs of 1200 ms including the baseline window of 200 ms before stimulus onset. We conducted an independent component analysis (ICA) to identify and remove ocular artifacts and generic discontinuities based on visual inspection and the ADJUST algorithm (Mognon et al. 2011).

To ensure that experimental conditions of the switching task were evenly distributed within each participant, we identified each participant’s experimental condition with the lowest number of trials and randomly drew the same number of trials from each of the other three experimental conditions. For example, when a participant had only 60 experimental trials in the odd/even-repeat condition, 60 trials each from the other three experimental conditions were randomly drawn to balance task demands. Subsequently, we calculated the ERP for each participant by averaging across trials and experimental conditions.

One participant’s EEG data set was lost for technical reasons, resulting in a final sample of 84 persons for electrophysiological analyses.

##### Preprocessing and Time-Frequency Decomposition of Electrophysiological Data

For the time frequency analyses, most of the preprocessing steps were equal to the ERP preparation. However, data were segmented into longer epochs of 4000 ms, starting 2000 ms before the onset of the fixation cross. Also, identical to the sample composition for ERP analyses, for time-frequency analyses the total sample size consisted of *N* = 84 participants.

Time frequency decomposition was performed with complex Morlet wavelets with frequencies ranging from 1 to 20 Hz in 20 linearly spaced steps. To specify the width of the Gaussian distribution, the number of *n* cycles was set to 4. This was chosen to provide a good trade-off between temporal and frequency resolution. Decibel-normalized alpha power was calculated for each participant in the time window from 1000 to 200 ms before the onset of the fixation cross as the mean power of the frequency bands between 8 to 12 Hz recorded at parieto-occipital electrode sites. This time window was chosen to examine variations in alpha power in an attentionally undemanding phase (within the inter-trial interval) before an imperative stimulus appears, which catches participants’ attentional focus back to the task at hand. To measure an internally directed attentional focus before the fixation cross was presented, the baseline window for inter-trial alpha power was set between 700 ms and 1000 ms after fixation cross onset. This allowed us to contrast alpha power of an attentionally undemanding phase to an attentionally focused phase. Decibel-normalized theta power was calculated for each participant in the time window from 0 to 500 ms after fixation cross onset as the mean power of the frequency bands between 4 to 7 Hz at fronto-central electrodes sites to examine differences in theta power after an imperative stimulus appeared and attentional resources had to be allocated. Theta power was averaged across frequencies and fronto-central electrode sites. The baseline window for task-evoked theta power was set between 1000 ms and 200 ms before the fixation cross was presented to assess attention-allocation following the presentation of the imperative stimulus. We selected the time-windows for both time-frequency domains based on findings of Arnau et al. (2020) who analyzed data from a subsample of Study 1.

##### Analyses of the Worst Performance Rule

In this study the WPR was examined with multilevel models based on the recommendations by Frischkorn et al. (2016). We were interested to test differences in covariances and correlations. Therefore, we followed the recommendations by Frischkorn et al. (2016) and used unstandardized as well as standardized coefficients for multilevel analyses to examine the increase of the magnitude in covariances and correlations between RT and intelligence across the RT distribution.

To get the full information of the whole RT distribution, we applied trial-by-trial analyses. To evaluate differences in the relations of intelligence and RT between faster and slower responses, we used the ascending number of the sorted trials to predict increases in RTs from the fastest to slowest trials. We included individual differences in intelligence as a between-subject predictor. A significant interaction in the multilevel model between trial number and intelligence would indicate that the relationship between RTs and intelligence changed across the RT distribution. In particular, the WPR implies a stronger negative relationship between RTs and intelligence in slower compared to faster trials. This was our baseline model.

To evaluate the effects of attentional lapses on response behavior in an ongoing task and their moderating implications on the WPR, we controlled for different combinations of attentional lapses indicators (behavioral, self-report, and electrophysiological measures). Therefore, we regressed the RTs for each corresponding sorted trial on these indicators. Afterwards we used the residuals of this regression as a new dependent variable. We then employed a stepwise procedure to test if controlling for attentional lapses reduced or removed the WPR. First, we tested if we still found a significant WPR after controlling for individual differences in attentional lapses. For this purpose, we again applied our baseline model, but instead of raw RTs, we used the residualized RTs as the new dependent variable. A non-significant WPR interaction between trial number and intelligence indicated a possible reduction of the slope of the WPR by attentional lapses. Because the difference between a significant and a non-significant result is not necessarily significant (Gelman and Stern 2006), we conducted further multilevel analyses to confirm this decrease statistically. For this purpose, we modified the multilevel models and included a dummy-coded within-subjects level-2 factor “control”. This factor indicated whether participants’ RTs were controlled for individual differences in attentional lapses (control = 1) or not (control = 0). If the interaction of trial number and intelligence changed as a function of this control factor—that is, if the three-way interaction between intelligence, trial number, and the control factor was significant—this would indicate that the size of the WPR changed after controlling for attentional lapses. We then used model comparisons based on the Akaike information criterion (AIC; Akaike 1998) to formally check if the introduction of this three-way-interaction (between the level-1 factor trial number, the level-2 factor control, and the between-subjects factor intelligence) improved substantially the model fit. Differences > 10 in AIC would indicate substantial differences in model fits (Burnham and Anderson 2002). For all analyses, we report degrees of freedom rounded to the nearest integer in case of non-integer numbers.

### 2.2. Results

The preprocessed data supporting the findings of Study 1 and the code for the statistical analysis used in this manuscript are available via the Open Science Framework (https://osf.io/5pafg/, accessed on 23 December 2021). Access to raw data of Study 1 will be granted upon request.

#### 2.2.1. Descriptive Results

For descriptive statistics of all variables see Table 1. All variables showed acceptable to very good reliabilities, estimated with Spearman-Brown corrected odd-even correlations or Cronbach’s alpha. Sample sizes differed slightly between the behavioral and the electrophysiological covariates, because EEG data from one participant were lost due to a technical error. For the correlations between all variables see Table 2. The closer the trial numbers were to each other, the higher their RTs were related. 

#### 2.2.2. Descriptive Analyses of Covariance and Correlation Patterns over the RT Distribution

On a descriptive level, we found increases of the magnitude in covariation from the fastest, *cov trial.1* = −10.93, to the slowest trials, *cov trial.340* = −83.01, as well as increases in the magnitude of negative correlations, *r trial.1* = −.14, and *r trial.340* = −.31. The magnitude in covariances from the fastest to the slowest trial increased monotonically (see Figure 3A), whereas the correlations peaked in their magnitude after approximately 85 percent of the trials (maximum correlation: *r trial.346* = −.31). Afterwards, the magnitude of correlations decreased again (see Figure 3B). This right tail of the RT distribution is particularly interesting, because it reveals a simultaneous increase in covariations and a decrease in correlations in the slowest 15 percent of RT distribution. Together, this pattern of results indicates that the inter-individual variance in RTs increased substantially in the right tail of the RT distribution, for unknown reasons, without an accompanying increase in the relationship between RTs and intelligence. Because this pattern of results was highly surprising and violates the core prediction of the WPR to observe a monotonic increase in both covariances and correlations across the whole RT distribution, we excluded the slowest 15 percent of the trials from all further analyses. However, we will discuss this unexpected finding and its implications in the General Discussion.

#### 2.2.3. The Worst Performance Rule with Unstandardized Coefficients (Covariances)

We analyzed the data with multilevel analyses to test if covariances between RT and intelligence revealed a significant worst performance pattern from faster to slower trials (Table 3). This analysis revealed a significant main effect of intelligence, *b* = −44.18, *t*(85) = −2.77, *p* = .007, which indicated that more intelligent participants showed faster RTs than less intelligent ones. Moreover, we found a significant worst performance interaction between intelligence and trial number, *b* = −14.93, *t*(85) = −2.85, *p* = .005, which confirms the presence of a statistically robust increase of the magnitude in covariances between RTs and intelligence over the RT distribution in our data. The worst performance interaction showed a medium effect size of η²part = 0.09. This result can be interpreted as follows: In the central trial with the sorting number of 170 (it corresponds to trial number 0 after rescaling between −2 and 2), a participant with an intelligence test score one *SD* above the mean was about 44 ms faster in their responses than an average intelligent participant. However, in a slow trial (trial number 255, which corresponds to the rescaled trial number 1), the same participant was even 59 ms faster than an average intelligent participant, whereas their RT difference was relatively negligible in a fast trial (trial number 85, which corresponds to the rescaled trial number −1), with only a difference of about 29 ms. Taken together, our baseline model indicated a significant WPR on the level of covariances. In the next steps we examined the influences of several behavioral and self-reported measures of attentional lapses on the unstandardized WPR.

#### 2.2.4. Do Individual Differences in Behavioral and Self-Reported Measures of Attentional Lapses Account for the WPR with Unstandardized Coefficients (Covariances)

**Figure 4:**
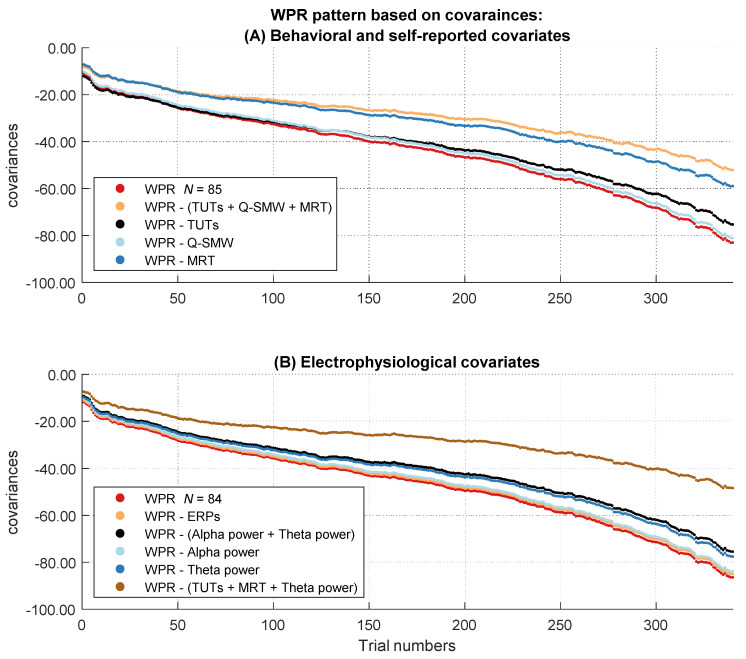
Course of the covariances over the RT distribution before and after controlling for the influence of the attentional lapses covariates. The figure describes the worst performance pattern in covariances before (red lines) and after (other lines) the different covariates or their combinations were partialized out of the RT variable (labeled in the boxes on the side of the dashes in the figure legend). (**A**) shows the results of the behavioral and self-reported covariates in the full sample of *N* = 85. (**B**) shows the results of the electrophysiological covariates in the subsample of *N* = 84.

In the next step, we analyzed if the increase of the magnitude in covariation disappeared after controlling for behavioral and self-report measurements of attentional lapses (TUT rates, Q-SMW scores, RT variability in the MRT). Therefore, we controlled participants’ RTs for individual differences in attentional lapses. Afterwards, we tested in multilevel analyses if the covariances between RT and intelligence still revealed a significant worst performance pattern. Figure 4A shows the descriptive course of covariances between RT and intelligence over the sorted trials before and after controlling for behavioral and self-reported attentional lapses. The two-way interaction between trial number and intelligence was no longer significant after controlling for individual differences in behavioral and self-report measures of attentional lapses, *b* = −8.88, *t*(85) = −1.82, *p* = .073 (Table S1 in the Supplementary Materials). 

To test if the changes in the WPR after controlling for individual differences in attentional lapses were significant, we merged both data sets (not controlled and controlled for attentional lapses) together and introduced a dummy-coded level-2 factor named “control” for moderation analyses in our multilevel model (Table 4). Hence, the RT variable in this multilevel model either reflected raw RTs or the residuals of those RTs after controlling for the influence of the covariates. A significant interaction between intelligence, trial number, and the control factor indicated that the increase of the magnitude in covariation between intelligence and RTs from faster to slower trials changed significantly after controlling for attentional lapses. This three-way interaction between intelligence, trial number, and the control factor was indeed significant, *b* = 6.05, *t*(57630) = 25.70, *p* < .001. The effect size of the three-way interaction revealed a small effect, η²part = 0.01. 

To additionally determine whether including the three-way interaction significantly improved the model fit, we compared our model to a more parsimonious model without this three-way interaction. Model comparison revealed a significantly better fit for the model with the three-way interaction as indicated by smaller AIC values, Δ_AIC_ = 655. Taken together, these results indicate that the behavioral and self-reported attentional lapses covariates together explained substantial parts of the worst performance pattern in covariances. To assess more specifically which behavioral and self-reported indicator of attentional lapses was most relevant, we examined the specific influence of each behavioral and self-report covariate on the WPR using the same procedure.

##### Task-Unrelated Thoughts (TUTs)

If we controlled for TUTs, we still observed a significant worst performance interaction in the baseline model, *b* = −12.98, *t*(85) = −2.55, *p* = .013 (Table S2 in the Supplementary Materials). Nevertheless, the significant three-way interaction between intelligence, trial number, and the control factor in the full model indicated that TUTs had an effect on the worst performance pattern, *b* = 1.95, *t*(57630) = 12.24, *p* < .001 (Table S3 in the Supplementary Materials). Model comparison revealed a better fit for the full model with the three-way interaction, Δ_AIC_ = 147. The effect size was very small, η²part = 0.00. Taken together, these results indicate that self-reported TUTs accounted for small parts of the WPR in covariances.

##### Questionnaire of Spontaneous Mind Wandering (Q-SMW)

If we controlled for Q-SMW scores, we still observed a significant worst performance interaction in the baseline model, *b* = −14.73, *t*(85) = −2.81, *p* = .006 (Table S4 in the Supplementary Materials). The three-way interaction between intelligence, trial number, and the control factor in the full model was not significant, indicating that the worst performance pattern did not change after controlling for Q-SMW scores, *b* = 0.20, *t*(57630) = 1.34, *p* = .179, η²part = 0.00 (Table S5 in the Supplementary Materials). Consequently, model comparison did not indicate a better fit for the full model with the three-way interaction, Δ_AIC_ = 0. Taken together, these results indicate that Q-SMW scores did not contribute to the WPR in covariances.

##### Metronome Response Task (MRT)

After controlling for the RT variability in the MRT, we still observed a significant worst performance interaction in the baseline model, *b* = −10.57, *t*(85) = −2.09, *p* = .039 (Table S6 in the Supplementary Materials). The significant three-way interaction between intelligence, trial number, and the control factor in the full model indicated a smaller worst performance pattern after controlling for RT variability in the MRT, *b* = 4.36, *t*(57630) = 19.60, *p* < .001 (Table S7 in the Supplementary Materials). Also, model comparison revealed a better fit for the full model with the three-way interaction, Δ_AIC_ = 380. Effect size estimation revealed a small effect, η²part = 0.01. Taken together, these results indicate that RT variability in the MRT accounted for some parts of the WPR in covariances.

#### 2.2.5. Do Individual Differences in Electrophysiological Measures of Attentional Lapses Account for the WPR with Unstandardized Coefficients (Covariances)

Figure 4B shows the descriptive course of covariances between RT and intelligence over the sorted trials before and after controlling for the electrophysiological covariates representing attentional lapses. The baseline multilevel model indicated a significant interaction between trial number and intelligence in this subset, *b* = −15.21, *t*(84) = −2.88, *p* = .005, η²part = 0.09 (Table S8 in the Supplementary Materials). 

##### ERP Analyses

If we controlled for individual differences in mean occipital P1 and mean centro-parietal P3 amplitudes, the two-way interaction between trial number and intelligence remained significant in the baseline model, *b* = −14.99, *t*(84) = −2.84, *p* = .006 (Table S9 in the Supplementary Materials). We observed no significant three-way interaction between intelligence, trial number, and the control factor in the full model, indicating that the size of the WPR did not change after controlling for the ERP mean amplitudes, *b* = 0.22, *t*(56952) = 1.42 *p* = .156, η²part = 0.00 (Table S10 in the Supplementary Materials). Consequently, model comparison did not reveal a better fit for the full model with the three-way interaction, Δ_AIC_ = 1. Taken together, these results indicate that the mean occipital P1 amplitude and the mean parietal P3 amplitude did not account for the WPR in covariances.

##### Time-Frequency Analyses

If we controlled for individual differences in alpha and theta power, the two-way interaction between trial number and intelligence remained significant in the baseline model, *b* = −13.14, *t*(84) = −2.55, *p* = .013 (Table S11 in the Supplementary Materials). Still, the significant three-way interaction between intelligence, trial number, and the control factor in the full model indicated a decrease in the worst performance pattern after controlling for alpha and theta power, *b* = 2.06, *t*(56952) = 9.98 *p* < .001 (Table S12 in the Supplementary Materials). Model comparison revealed a better fit for the full model with the three-way interaction, Δ_AIC_ = 98. However, this effect was very small, η²part = 0.00. Taken together, these results indicate that the time-frequency covariates accounted for small parts of the WPR in covariances. To detect the unique influence of the two different time-frequency covariates on the WPR, we estimated the models for both covariates separately.

##### Alpha-Power

After controlling for individual differences in alpha power, the two-way interaction between trial number and intelligence remained significant in the baseline model, *b* = −14.96, *t*(84) = −2.87, *p* = .005 (Table S13 in the Supplementary Materials). More importantly, there was no significant three-way interaction between intelligence, trial number, and the control factor in the full model, indicating that the size of the WPR did not change after controlling for alpha power, *b* = 0.24, *t*(56952) = 1.41 *p* = .159, η²part = 0.00 (Table S14 in the Supplementary Materials). Model comparison did not reveal a better fit for the full model with the three-way interaction, Δ_AIC_ = 0. Taken together, these results indicate that individual differences in inter-trial alpha power did not account for the WPR in covariances.

##### Theta-Power

After controlling for individual differences in theta power, the two-way interaction between trial number and intelligence remained significant in the baseline model, *b* = −13.48, *t*(84) = −2.57, *p* = .012 (Table S15 in the Supplementary Materials). The significant three-way interaction between intelligence, trial number, and the control factor in the full model indicated a significant change of the worst performance pattern after controlling for theta power, *b* = 1.72, *t*(56952) = 9.73, *p* = .001 (Table S16 in the Supplementary Materials). Model comparison also showed a better fit for the model with the three-way interaction, Δ_AIC_ = 96, but the effect size of the three-way interaction was very small, η²part = 0.00. Taken together, these results indicate that theta power accounted for small parts of the WPR in covariances. 

##### The Combined Effect on the Unstandardized Worst Performance Pattern of All Predictors with a Substantial Contribution (TUTs, MRT, Theta-Power)

After controlling for individual differences in covariates with a unique contribution to the explanation of the WPR, we examined their combined influence. The two-way interaction between trial number and intelligence was no longer significant in the baseline model, *b* = −7.76, *t*(84) = −1.59, *p* = .116 (Table S17 in the Supplementary Materials). The significant three-way interaction between intelligence, trial number, and the control factor in the full model indicated a substantial change of the worst performance pattern after controlling for all three predictors, *b* = 7.45, *t*(56952) = 28.68 *p* < .001 (Table S18 in the Supplementary Materials). Model comparison revealed a significantly better fit for the full model with the three-way interaction, Δ_AIC_ = 815. The estimation of the effect size indicated a small effect, η²part = 0.01. All in all, these results indicate that TUT rates, variability in the MRT, and theta power together fully explained the worst performance pattern in covariances.

#### 2.2.6. The Worst Performance Rule with Standardized Coefficients (Correlations)

On the level of correlations, we did not find a significant worst performance pattern in the baseline multilevel model, *b* = −0.02, *t*(85) = −1.10, *p* = .276 (Table S19 in the Supplementary Materials). We also did not find a significant worst performance interaction, *b* = −0.02, *t*(84) = −0.91, *p* = .359, in the subset with psychophysiological covariates (Table S28 in the Supplementary Materials). The worst performance interaction revealed a small effect size of η²part = 0.01. We observed a small descriptive increase in the magnitude of negative correlations from the first to the last trial of Δ*_r_* = .08 (Figure 3B).

#### 2.2.7. Do Individual Differences in Behavioral and Self-Reported Measures of Attentional Lapses Account for the WPR with Standardized Coefficients (Correlations)

**Figure 5:**
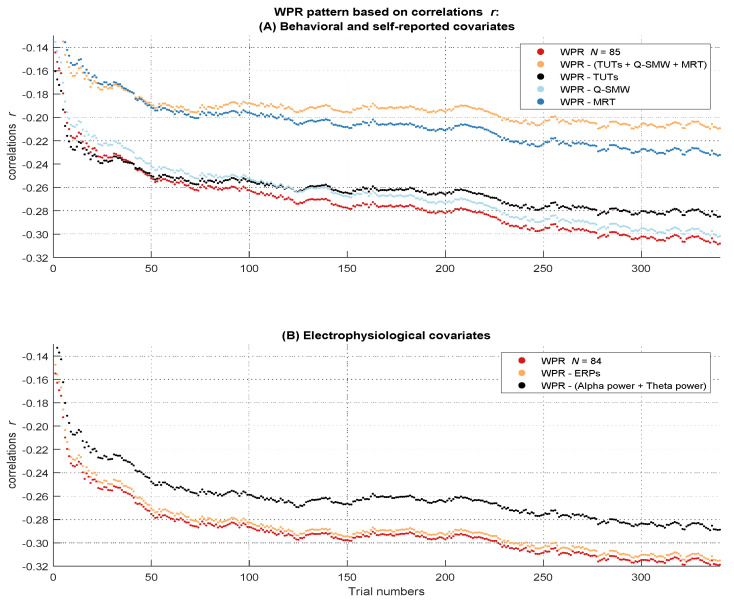
Course of the correlations over the RT distribution before and after controlling for the influence of the attentional lapses covariates**.** The figure describes the worst performance pattern in correlations before (red lines) and after (other lines) the different covariates or their combinations were partialized out of the RT variable (labeled in the boxes on the side of the dashes in the figure legend). (**A**) shows the results of the behavioral and self-reported covariates in the full sample of *N* = 85. (**B**) shows the results of the electrophysiological covariates in the subsample of *N* = 84.

Because there was no significant worst performance interaction in the baseline multilevel model with standardized coefficients and we found no suppressor effect of the covariates on this interaction, we will not report the baseline models without the effect of any covariates (they can be found in Tables S20, S22, S24, S26, S29 and S31 in the Supplementary Materials). The significant three-way interaction between intelligence, trial number, and the control factor in the full model indicated a change in the worst performance pattern after controlling for the behavioral and self-reported covariates, *b* = 0.01, *t*(57630) = 8.70, *p* < .001 (Table S21 in the Supplementary Materials). Model comparison revealed a better fit for the full model with the three-way interaction, Δ_AIC_ = 73. However, the effect size of η²part = 0.00 suggested that this effect was very small. Taken together, the behavioral and self-reported attentional lapses covariates together explained very small parts of the (not significant) worst performance pattern in correlations. To assess more specifically which behavioral and self-reported indicator of attentional lapses was most relevant for this effect, we additionally examined the individual influence of each of these covariates on the WPR in correlations by using the already known procedure (Figure 5A).

##### Task-Unrelated Thoughts (TUTs)

The significant three-way interaction between intelligence, trial number, and the control factor in the full model indicated a smaller worst performance pattern after controlling for TUTs, *b* = 0.01, *t*(57630) = 9.49, *p* < .001 (Table S23 in the Supplementary Materials). Model comparison revealed a better fit for the full model with the three-way interaction, Δ_AIC_ = 88. The effect size of η²part = 0.00 indicated a very small effect of TUTs on the WPR. Taken together, these results indicate that self-reported TUTs accounted for a very small part of the WPR in correlations.

##### Questionnaire of Spontaneous Mind Wandering (Q-SMW)

The three-way interaction between intelligence, trial number, and the control factor in the full model was not significant, indicating that the worst performance pattern did not change after controlling for Q-SMW scores, *b* = 0.00, *t*(57630) = 1.39, *p* = .165, η²part = 0.00 (Table S25 in the Supplementary Materials). Consequently, model comparison did not indicate a better fit for the full model with the three-way interaction, Δ_AIC_ = 0. Taken together, these results indicate that Q-SMW scores did not contribute to the WPR in correlations.

##### Metronome Response Task (MRT)

The significant three-way interaction between intelligence, trial number, and the control factor in the full model indicated a smaller worst performance pattern after controlling for RT variability in the MRT, *b* = 0.00, *t*(57630) = 3.47, *p* < .001 (Table S27 in the Supplementary Materials). Model comparison revealed a better fit for the full model with the three-way interaction, Δ_AIC_ = 10. We found only a very small effect of the MRT on the WPR, η²part = 0.00. Taken together, these results indicate that RT variability in the MRT accounted only for a very small part of the WPR.

#### 2.2.8. Do Individual Differences in Electrophysiological Measures of Attentional Lapses Account for the WPR with Standardized Coefficients (Correlations)

##### ERP Analyses

There was no significant three-way interaction between intelligence, trial number, and the control factor in the full model, indicating that the size of the WPR did not change if we controlled for the ERP amplitudes, *b* = 0.00, *t*(56952) = −0.32 *p* = .749, η²part = 0.00 (Table S30 in the Supplementary Materials). Consequently, model comparison did not show a better fit for the full model with the three-way interaction, Δ_AIC_ = 2. Taken together, these results indicate that mean occipital P1 and centro-parietal P3 amplitudes could not account for the WPR in correlations (see Figure 5B).

##### Time-Frequency Analyses

The three-way interaction between intelligence, trial number, and the control factor in the full model indicated that there was no difference in the worst performance pattern after controlling for the combined influence of mean alpha and theta power, *b* = 0.00, *t*(56952) = 1.17 *p* = .243, η²part = 0.00 (Table S32 in the Supplementary Materials). Model comparison also did not show a better fit for the full model with the three-way interaction, Δ_AIC_ = 1. Taken together, these results suggest that the time-frequency covariates could not account for the WPR in correlations.

### 2.3. Discussion

Our findings provided some evidence for the attentional lapses account of the worst performance rule. We found a significant increase in the magnitude of covariances between intelligence and RTs from the fastest to the slowest RTs (i.e., a WPR). This increase was less strong when we controlled for inter-individual differences in several of the self-reported attentional lapses measures. Notably, after combining different attentional lapses measures and controlling for these, the WPR disappeared. Thus, inter-individual differences in the propensity of attentional lapses did fully explain the WPR in the present data set on the level of covariances. Nevertheless, it has to be stressed that the combined effect of attentional lapses on the WPR was very small (η²part = 0.01). It is possible that we were only able to detect this small effect of attentional lapses on the WPR due to the high statistical power of the multi-level account and the trial-by-trial analyses.

However, there was no significant WPR on the level of correlations. Nevertheless, descriptively, there was still an increase in the negative correlations with a magnitude of about *r* = −.08, which is consistent with former research investigating the WPR on a descriptive level (e.g., Fernandez et al. 2014). Again, the increase in the magnitude was reduced after controlling for self-reported attentional lapses but the present data do not address the question of the extent to which attentional lapses can account for the WPR on the level of correlations, as we did not find a significant WPR on that level. Apparently, the statistical power was rather low for a detection of an effect with the magnitude of the WPR on the correlational level. Thus, one reason for why we did not observe a significant WPR on the correlational level probably was our somewhat low sample size. We tackled this problem with our second study.

#### 2.3.1. Influence of Covariates on the WPR in Covariances

Different covariates of attentional lapses showed a significant influence on the WPR and controlling for them reduced the increasing magnitude in covariances. In particular, controlling for self-reported attentional lapses led to a reduction of the worst performance pattern and provided evidence for the attentional lapses account. However, we found some unexpected relations between self-reported attentional lapses and participants’ mean RTs/RT variability as well as between TUTs and intelligence. These correlations between the measures were not in line with former findings and contrary to theoretical predictions. In detail, individuals who reported more attentional lapses, measured by TUTs, showed faster RTs and less RT variability as well as higher intelligence test scores in our data. The attentional lapses account, in contrast, states that individuals with lower cognitive abilities should experience more attentional lapses and should be slower in their responses. Also, individuals with lower cognitive abilities should show more variability of their responses within a certain task. Previous studies showed typically the opposite direction of correlations compared to our findings (e.g., Kane et al. 2016; McVay and Kane 2009, 2012; Randall et al. 2014; Robison et al. 2020; Welhaf et al. 2020). Possible reasons for these surprising correlations may be the size or composition of our sample and will be discussed below.

Besides self-reported attentional lapses, one of the objective measures (i.e., the RT variability in the MRT) also contributed to the explanation of the WPR. The MRT is typically used as an alternative, more objective measure of attentional lapses (Anderson et al. 2021; Seli et al. 2013). However, Figure 4 and Figure 5 show that the MRT explained not only the slope of the WPR but also large parts of the covariances and correlations over the whole RT distribution. It is plausible that the MRT and the assigned decision making task for assessing the WPR possess some overlaps. Performances in both tasks were measured via RTs, which are determined by different processes, such as the encoding of stimuli and the preparation of the motor response. Thus, controlling for MRT variability in our reaction time task means that we also have controlled for some variance resulting from these processes. This could be the reason for the similar reduction of the covariances and correlations over the whole RT distribution after controlling for the MRT.

It must be noted that several of our covariates did not contribute to the WPR. This was especially surprising in case of the Q-SMW, as the underlying construct (i.e., mind wandering tendencies) are supposed to be strong predictors of attentional lapses. In the present sample, questionnaire scores were moderately correlated with self-reported attentional lapses during the task. This is consistent with earlier studies showing that mind wandering trait questionnaires predict the frequency with which attentional lapses are experienced while participants work on an experimental task (Mrazek et al. 2013; Schubert et al. 2020). Mind wandering is, however, a broad construct covering a range of attentional phenomena. This may explain why the thought-probing measure of attentional lapses but not the global mind wandering questionnaire explained parts of the WPR.

On the electrophysiological level, the mean amplitudes of the lateral-occipital P1 and the centro-parietal P3 as well as mean parieto-occipital inter-trial alpha power showed no effects on the WPR. Only mean stimulus-evoked fronto-central theta power changed the course of covariances over the RT distribution. It is surprising that the electrophysiological covariates did not change the worst performance pattern, because former studies found relations of the centro-parietal P3 to TUTs (Kam and Handy 2013; Smallwood et al. 2008), to sustained attention (O’Connell et al. 2009), and to the allocation of cognitive resources (Allison and Polich 2008; Kok 2001). Likewise, former studies demonstrated that attentional lapses and neural processing of stimuli via the occipital visual P1 are related (Baird et al. 2014; Kam et al. 2011). Also, inter-trial alpha power, which reflects internally directed mental states and which was shown to be strongly predictive for the experience of attentional lapses (Arnau et al. 2020), could not explain the WPR. Altogether, it seems that the chosen electrophysiological covariates did not account for the WPR, except for the very small effect of mean theta power.

#### 2.3.2. Influence of Covariates on the WPR in Correlations

Self-reported attentional lapses and intra-individual RT variability of the MRT accounted for the WPR on the level of correlations. Descriptively it seemed that the MRT explained large parts of the correlations, but the effect of the MRT on the WPR in the multilevel models was slightly smaller compared to the effect of self-reported attentional lapses. This underlines the just discussed proposition that the MRT accounts for RT properties unrelated to the slope of the WPR. In contrast to the analyses of the covariances, on the level of correlations mean fronto-central theta power could not account for the worst performance pattern. Again, all other covariates revealed no effect on WPR.

#### 2.3.3. Low Correlation and Unpredicted Correlations with Attentional Lapses Measures

There were hardly any correlations between different attentional lapses measures or their psychophysiological correlates. It is well known that individual occurrences of attentional lapses depend on personal and context-related variables, which means that the construct of attentional lapses shows a multiverse structure (Robison et al. 2020).

Nevertheless, beyond the multiverse structure of the attentional lapses construct, the low correlations should also be considered as challenging for attentional lapses research. The absence of relations between different attentional lapses measures raises the question of construct validity. If we try to capture a certain ability or a state of attention with a multimethod approach, these measures should all reflect a common latent construct. This assumption should be empirically reflected in—at least—small correlations between those measures. A problem of attentional lapses research is the vague definition of attentional lapses, which leads to more degrees of freedom in its operationalization. Future research should further examine the construct validity of attentional lapses. 

In contrast to former findings (e.g., Kane et al. 2016; McVay and Kane 2009, 2012; Randall et al. 2014; Robison et al. 2020; Welhaf et al. 2020) and to predicted relations, we found that TUTs and cognitive abilities as well as RT and RT variability measures were not related or that their correlations pointed in the unpredicted direction.

#### 2.3.4. Interim Conclusion

Generally, each attentional lapses indicator explained unique parts of the worst performance pattern. When we examined the common influence of different attentional lapses covariates on the WPR, the WPR disappeared fully on the level of covariances (Figure 4). On a descriptive level, we also observed a clear change in the pattern of correlations from the fastest to the slowest RTs (Figure 5). Our findings are in line with the idea that attentional lapses have different facets, which should be captured by different indicators (Robison et al. 2020). Due to the multiverse structure, measures of attentional lapses do not need to converge (e.g., Mrazek et al. 2013; Schubert et al. 2020; Seli et al. 2013). We found the same pattern in our results with weak or absent correlations between the different measures of the attentional states (Table 2). This underscores the necessity of the multimethod approach, which we chose in the present study by assessing attentional lapses with self-reports, objective indicators, and psychophysiological measures to capture individual differences in this construct as comprehensively as possible, which is as a major advantage of our study.

Nevertheless, despite the clear descriptive worst performance pattern in correlations in our study and despite the recent meta-analysis by Schubert (2019), who reported robust evidence for the presence of the WPR, we did not find a significant WPR on the level of correlations. There are several possible explanations for this. First, the sample size in this study was small and consequently the statistical power was too low to detect a significant WPR in our multilevel models given the small effect size. Additionally, the multilevel approach, proposed by Frischkorn et al. (2016), considers the uncertainty in correlation estimates. In a small sample, the confidence intervals of the estimators are quite large, and therefore the differences in correlations may not have become significant in our analyses. A larger sample size would minimize the uncertainty in the estimators (Schönbrodt and Perugini 2013).

Second, the absence of the WPR may also be attributed to the heterogeneity of our sample. It is known that student samples differ in many psychological variables compared to general population or even representative samples (Hanel and Vione 2016). In addition, age may have affected participants’ response behavior in self-reported attentional lapses and RTs. For example, previous studies found fewer instances of attentional lapses in older people as compared to younger people (e.g., Arnicane et al. 2021; Frank et al. 2015; Krawietz et al. 2012; Maillet et al. 2018, 2020; Maillet and Schacter 2016). Furthermore, it is well established that older participants respond slower compared to younger participants (e.g., Verhaeghen and Salthouse 1997). As we have recruited an age-heterogeneous sample, age differences may have obscured our covariance structure. We found no evidence for an age-related decline in the frequency of reported attentional lapses in our sample (*r* = −.14, *p* = .201), but older participants showed slower responses (*r* = .26, *p* = .016).

Third, the measurement took place in a highly controlled laboratory situation. In order to achieve a clear measure of brain activity with the EEG, participants were individually seated in a shielded cabin so that any kind of noise was reduced to a minimum. Consequently, participants of our study probably experienced fewer distractions than in standard behavioral laboratory studies. It is possible that the special laboratory situation of our study influenced the occurrence and experience of attentional lapses and in consequence the magnitude of the WPR.

Because of the mentioned shortcomings of our first study (low power resulting from the small sample size, heterogeneity of the sample, and unexpected correlations between intelligence, RTs or RT variability and self-reported attentional lapses), we reanalyzed an already published data set with our approach to test whether the results and descriptive patterns would replicate in an independent larger and more homogenous student sample. In Study 2, we were particularly interested if we would find a significant WPR (and a reduction thereof when controlling for inter-individual differences in attentional lapses) on the correlational level when the statistical power was increased.

## 3. Study 2

### 3.1. Materials and Methods

To replicate our results in an independent sample, we reanalyzed the data set from two previously published studies by Kane et al. (2016) and Welhaf et al. (2020). From these previous studies it is already known that the correlations between TUTs, RTs, and intelligence are in accordance with expectations, which we consider an advantage of this data set. The data for Study 2 are available via the Open Science Framework. Use https://osf.io/9qcmx/ (accessed on 5 February 2021) to access the raw data and use https://osf.io/5pafg (accessed on 23 December 2021) to get access to additional data, which are not provided via the previous link.

#### 3.1.1. Participants

At three measurement occasions, Kane et al. (2016) recruited a total sample of 545 undergraduates, aged between 17 and 35 years, from the University of North Carolina at Greensboro and Minority-Serving state university. For the present analyses, the number of available data-sets differed between the tasks (arrow-flanker *N* = 481, letter-flanker *N* = 426, number-stroop *N* = 481, sustained attention to response task [SART] *N* = 486). In consequence of outlier analyses, different numbers of participants remained for each task (see Data Preparation below for specific information). We analyzed the data with the same analysis strategy as used in Study 1. The mean age of the analyzed subsample was 18.92 (*SD* = 1.91), 66.94 percent of the sample were female. Five participants did not disclose their gender.

#### 3.1.2. Materials

##### Sustained Attention Task (SART)

Participants had to press the space bar in go-trials (89% of 675 trials) and to withhold their response in no-go-trials (11% of 675 trials). Go-trials were indicated by words of the category “animals” and no-go trials were indicated by words of the category “vegetables”. We used RTs of go-trials as dependent variable, consistent with the analyses by Welhaf et al. (2020).

##### Letter-Flanker

Participants had to decide whether the presented target letter “F” appeared normally or backwards. The letter was presented amid six distractors on the horizontal line. In total, participants had to respond in 144 trials, which consisted of 24 neutral trials (the target letter was presented amid dots), 48 congruent trials (the target and the distractors were the same letters and pointed in the same direction), 24 trials of an incongruent condition (the target and the distractors were the same letters, but only five out of the six distractors pointed in the same direction as the target), 24 stimulus-response incongruent trials (the target and the distractors were the same letters but pointed in the opposite directions), and 24 stimulus-stimulus incongruent trials (the distractors consists of the letters “E” and “T”, which were additionally tilted by 90 and 270 degrees). We used the RTs of correctly solved congruent and neutral trials as dependent variable, consistent with the analyses by Welhaf et al. (2020).

##### Arrow-Flanker

Participants had to decide whether a centrally presented arrow pointed to the right or to the left. The arrow was presented amid four distractors on the horizontal line. In total participants had to respond in 192 trials, which consisted of 48 neutral trials (the target was presented amid dots), 48 congruent trials (the target and the distractors pointed in the same direction), 48 stimulus-response incongruent trials (the target and the distractors pointed in the opposite directions), and 48 stimulus-stimulus incongruent trials (the distractor arrows pointed upwards). We used the RTs of correctly solved congruent and neutral trials as dependent variable, consistent with the analyses by Welhaf et al. (2020).

##### Number-Stroop

In each trial, two to four digits were presented in a row. Participants had to count the quantity of presented digits, while they had to ignore their meaning. They responded by pressing one of three labeled keys. The condition could be congruent, if the quantity of presented digits was equal to their meaning (e.g., 4444 or 333), or incongruent, if the quantity of presented digits differed from their meaning (e.g., 2222 or 44). Twenty percent of the 300 trials were incongruent trials. We used the RTs of correctly solved congruent trials as dependent variable, consistent with the analyses by Welhaf et al. (2020).

##### Working Memory Capacity

In Study 2 we used WMC as an independent variable to measure cognitive abilities. This is unproblematic, because the WPR was also observed in the relations between RTs an WMC (McVay and Kane 2012; Schmiedek et al. 2007; Unsworth et al. 2010; Welhaf et al. 2020). Furthermore, WMC is highly related to intelligence (Conway et al. 2002; Kane et al. 2005; Kyllonen and Christal 1990; Oberauer et al. 2005) and therefore a suitable alternative measure of cognitive abilities beside intelligence. Moreover, individual differences in attentional lapses should account for individual differences in both WMC as well as intelligence (Kane et al. 2008; Shipstead et al. 2016). WMC was measured with six different tasks. Four of these tasks required maintaining serially presented memory items while participants had to repeatedly engage in an unrelated secondary task (Operation-Span, Sentence-Span, Symmetry-Span, and Rotation-Span). Participants’ responses were coded as correct if they recognized memory items in their correct serial position. The two remaining tasks measuring WMC required participants’ ability for updating previously memorized items (Running-Span-Task and Updating-Counters). Participants’ responses were coded as correct if they recognized the updated memory items. For more detailed information on the tasks, see Kane et al. (2016). We used the latent WMC scores calculates by Welhaf et al. (2020). These were estimated with confirmatory factor analyses and full information maximum likelihood was used to account for missing data when the factor scores were computed.

##### Online Thought-Probing Procedure

At each online thought probe, participants were asked: “What are you thinking about?” and had to answer by pressing one of eight keys which most closely matched their thought content. They could choose between: (1) The task—on-task thoughts; (2) Task experience/performance—thoughts about one’s own task performance; (3) Everyday things—thoughts about routine things; (4) Current state of being—thoughts about one’s own current physical or emotional state; (5) Personal worries—thoughts about one’s worries and concerns; (6) Daydreaming—fantastic thoughts, which are decoupled from reality; (7) External environment—thoughts about the immediate external environment; (8) Other—thoughts which do not fit in one of the other seven categories. Kane et al. (2016) as well as Welhaf et al. (2020) coded all answers of the categories 1 and 2 as on-task and all answers of the categories 3 to 8 as off-task thoughts (TUTs). We used the rate of these TUTs as a measure of attentional lapses. The attentional lapses covariate contained 45 thought probes from the SART, 20 from the Number-Stroop task, 20 from the Arrow-Flanker task, and 12 from the Letter-Flanker task, as well as 12 from an otherwise-not further reported and analyzed 2-back task.

#### 3.1.3. Data Preparation and Analyses

Within each task, we removed participants with fewer than 50 percent of correctly answered trials. In the next step, the two trials following thought probes, responses faster than 150 ms and slower as 3000 ms, incorrect responses, and trials of the non-analyzed conditions were discarded within each task. Afterwards, we removed all participants who showed higher logarithmical accuracy *z*-scores than 3 *SD*s from the sample mean within each task. After that, we conducted an intra-individual outlier analysis and discarded all trials with RTs that deviated more than 3 *SD*s from the mean of the intra-individual logarithmized RT distribution within each task. Finally, within each task, we removed the participants with higher mean RT *z*-scores than 3 *SD*s from the sample mean.

We sorted all of the remaining trials within each participant in each task in the ascending order according to their RTs. All participants with at least 60 remaining trials in the arrow-flanker task, 50 remaining trials in the letter-flanker task, 170 remaining trials in the number-stroop task, as well as at least 200 remaining trials in the SART were included to ensure a sufficient and comparable number of trials on the one hand and to minimize the number of participants with fewer trials who had to be excluded from the analyses on the other hand. In consequence of this minimal amount of trials criterion, we removed different numbers of participants within each task from further analyses. This led to a final sample of 463 participants in the arrow-flanker task (28 participants were removed as outliers), 416 participants in the letter-flanker task (10 participants were removed as outliers), 460 participants in the stroop task (21 participants were removed as outliers), and 441 participants in the SART (45 participants were removed as outliers). We used the middle trials of each participant’s RT distribution in each task and removed the remaining trials symmetrically from both ends of the intraindividual distribution. Multilevel analyses were conducted in the same way as in Study 1. We included all of the four tasks in one model and added the task as an additional effect-coded level-3 factor. The factor levels of the task-factor were contrasted to the SART. All multilevel models were estimated using the “nlminb” optimizer, except for the two full models in which the WPR was controlled for TUT rates, because those two models only converged with the “L-BFGS-B” optimizer algorithm.

### 3.2. Results

#### 3.2.1. Descriptive Analyses

Descriptive statistics are shown in Table 5 and the correlations between all relevant variables are shown in Table 6. Mean RTs as well as RT variability of the four different tasks were highly correlated. In contrast to Study 1, the correlations between TUTs and RTs, TUTs and RT variability, as well as between TUTs and cognitive abilities (in this case WMC) pointed in the hypothesized directions. For WMC, reliability estimation across the working memory tasks revealed an acceptable internal consistency with Cronbach’s α= .78.

**Figure 6:**
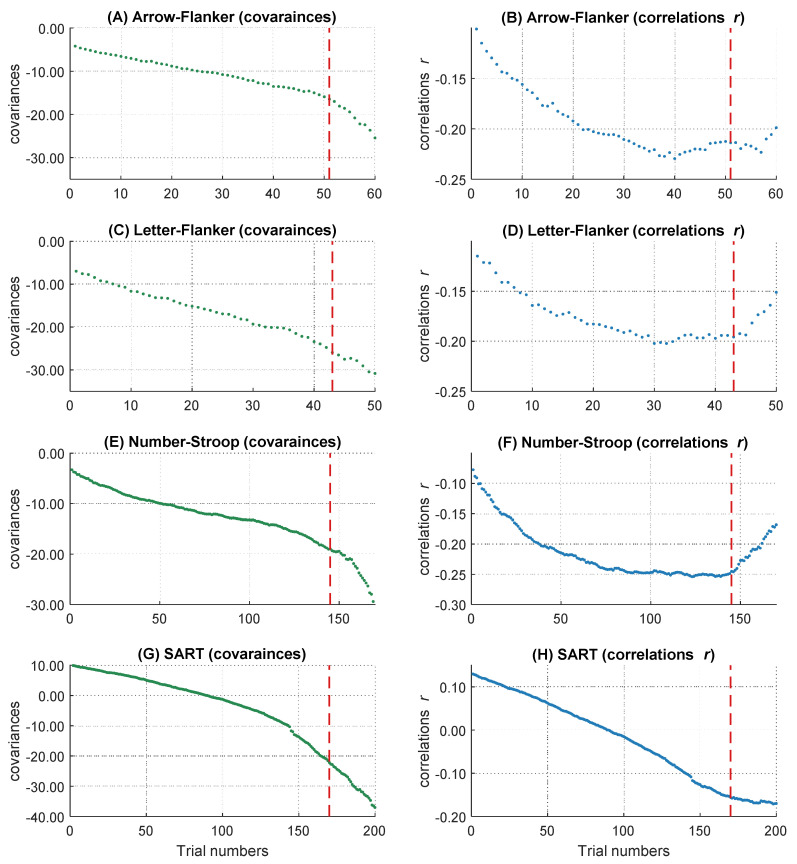
The increasing magnitude of negative correlations and covariances over RT distributions. The courses of the covariances in the four different tasks are shown on the left side (**A**,**C**,**E**,**G**). The courses of the correlations in the four different tasks are shown on the right side (**B**,**D**,**F**,**H**). The dashed lines represent the 85 percent thresholds. Only the left parts of the dashed lines were analyzed in the following multi-level analyses.

Over the RT distributions, we found the same pattern of correlations in most of the four tasks as we did in Study 1. After about 85 percent of the selected range of the RT distributions, the negative increases in the magnitude of the covariances accelerated, whereas the magnitude of the negative correlations decreased at this point (Figure 6). These descriptive findings were consistent over the different tasks and replicated our unexpected results from Study 1. For the comparability to the results of Study 1, we only analyzed the fastest 85 percent of each participant’s trials. Every participant contributed 51 trials from the arrow-flanker task, 43 trials from the letter-flanker task, 145 trials from the number-stroop task, and 170 trials from the SART to the multilevel models. Again, in each task, we centered the data to participants’ central trials and rescaled the trial numbers between −2 and 2. 

#### 3.2.2. The Worst Performance Rule with Unstandardized Coefficients (Covariances)

On the level of unstandardized coefficients, the baseline multilevel model indicated a significant interaction between trial number and WMC, *b* = −4.46, *t*(496) = −6.53, *p* < .001 (Table S33 in the Supplementary Materials). The worst performance interaction revealed a medium effect size of η²part = 0.08. There were significant interactions between the factor task and the worst performance effect (interaction with arrow-flanker task: *b* = 1.31 *t*(182674) = 5.14, *p* < .001; no interaction with letter-flanker task: *b* = 0.17, *t*(182657) = 0.61, *p* =.543; interaction with number-stroop task: *b* = 1.18, *t*(182711) = 6.28, *p* < .001), suggesting that the strength of the WPR varied between tasks. Separate follow-up analyses for each of the four tasks revealed that a significant worst performance interaction was present in each of the four tasks (all *p*s < .001).

**Figure 7:**
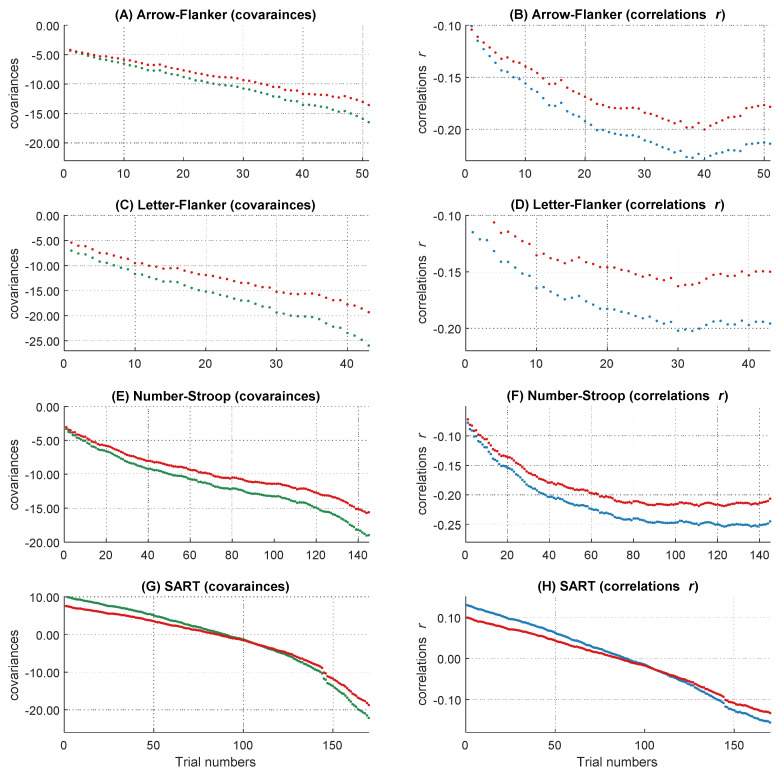
Course of the covariances and correlations over the RT distributions before and after controlling for the influence of the attentional lapses covariates. The courses of the covariances in the four different tasks are shown on the left side (**A**,**C**,**E**,**G**). The courses of the correlations in the four different tasks are shown on the right side (**B**,**D**,**F**,**H**). The figure describes the worst performance pattern before (green and blue lines) and after (red lines) the TUTs covariate were partialized out of the covariance.

After controlling for individual differences in attentional lapses, we still observed a significant two-way interaction between trial number and WMC in the baseline model, *b* = −3.44, *t*(496) = −5.20, *p* < .001 (Figure 7 left side, Table S34 in the Supplementary Materials). The significant three-way interaction between WMC, trial number, and the control factor in the full model indicated a small but significant change of the worst performance pattern after controlling for attentional lapses, *b* = 0.94, *t*(365374) = 5.07, *p* < .001 (Table S35 in the Supplementary Materials). Also, model comparison revealed a significantly better fit for the full model with the three-way interaction in comparison to a model without the three-way interaction, Δ_AIC_ = 38. Effect size estimation found a very small effect, η²part = 0.00. We found no effects of the task on the three-way interaction, which indicates that the influence of TUTs on the worst performance pattern was comparable for all tasks (all four-way interactions were not significant, all *p*s > .192). Taken together, these results indicate that TUTs accounted for a small part of the worst performance pattern in multilevel models with unstandardized coefficients. 

#### 3.2.3. The Worst Performance Rule with Standardized Coefficients (Correlations)

On the level of standardized coefficients, the baseline multilevel model indicated a significant interaction between trial number and WMC, *b* = −0.04, *t*(499) = −5.13, *p* < .001 (Table S36 in the Supplementary Materials). The worst performance interaction revealed a small effect size of η²part = 0.05. Again, we observed interactions between the task factor and the WPR (interaction with arrow-flanker task: *b* = 0.01, *t*(182643) = 3.28, *p* =.001; interaction with letter-flanker task: *b* = 0.02, *t*(182.633) = 5.41, *p* < .001; no interaction with number-stroop task: *b* = 0.00, *t*(182687) = 1.79, *p* =.074) but baseline models for all tasks showed significant worst performance interactions (all *p*s < .017).

After controlling for individual differences in attentional lapses, we still observed a significant two-way interaction between trial number and WMC in the baseline model, *b* = −0.03, *t*(499) = −4.05, *p* < .001 (Figure 7 right side, Table S37 in the Supplementary Materials). The significant three-way interaction between WMC, trial number, and the control factor in the full model indicated a small but significant change of the worst performance pattern after controlling for attentional lapses, *b* = 0.01, *t*(365373) = 3.42, *p* = .001 (Table S38 in the Supplementary Materials). Also, model comparison revealed a significantly better fit for the full model with the three-way interaction in comparison to a model without the three-way interaction, Δ_AIC_ = 19. Effect size estimation revealed an effect close to zero, η²part = 0.00. We found no effects of the task factor on the tree-way interaction, which indicates that the influence of TUTs on the worst performance pattern was comparable for all tasks (all four-way interactions were non-significant, all *p*s >.538). Taken together, these results indicate that TUTs accounted for very small parts of the worst performance pattern in the multilevel models with standardized coefficients (i.e., the WPR on the correlational level). 

### 3.3. Discussion

The results of Study 2 substantiated the main results of Study 1 that attentional lapses can explain the increasing magnitude of covariation of the WPR to a significant degree. The large sample size and the greater homogeneity of the sample (students; mean age = 18.92, *SD* = 1.91) are the main characteristics different from the Study 1 sample. In Study 2, we found a significant WPR in our multilevel models, both on the level of covariances as well as on the level of correlations. We found a larger effect of attentional lapses on the WPR on the level of covariances than on the level of correlational analyses. This confirms the choice of our strategy to examine the WPR on both levels and suggests that attentional lapses contribute not only to the relation between RTs and cognitive abilities, but also to the variance in RTs, which is independent of cognitive abilities. As in Study 1, the single measure of self-reported attentional lapses explained only a small part of the WPR. The WPR remained significant after controlling for TUTs, independent of whether we analyzed covariances or correlations. We therefore conclude that TUTs as the sole measurement of attentional lapses explain a small part of the worst performance pattern and substantial parts of the WPR remain unexplained.

Taken together, we found significant worst performance patterns in the data and replicated our multilevel model findings of Study 1 in a large and age-homogenous sample. As already known from former findings by Kane et al. (2016) and Welhaf et al. (2020), the relations between all variables (TUTs, WMC, RTs) were consistent with previous research and our predictions. Self-reported attentional lapses, measured as TUTs, explained some significant—albeit very small—part of the WPR. 

## 4. General Discussion

We analyzed two independent data sets and found support for Larson and Alderton’s (1990) idea that attentional lapses can explain parts of the worst performance pattern (Larson and Alderton 1990). According to our results, the contribution of attentional lapses to the WPR varied for each of the covariates and the effects of the single covariates appeared to be very small, which in turn led to a small but significant reduction of the WPR. Considering the multiverse structure of attentional lapses, we combined different covariates and examined their common influence on the WPR. The influence of self-reported attentional lapses and an objective attentional lapses indicator together led to a full explanation of the phenomenon. In Study 1, we found a significant reduction of the worst performance pattern in covariances and a significant decrease of the worst performance slope in correlations. To address statistical power issues and to replicate our findings, we applied the same analysis strategy in a larger independent student sample in Study 2. The results of this replication study were in line with our former findings and also statistically significant on both levels. Taken together, we found evidence for the attentional lapses account, which claims that the origin of the WPR is based on inter-individual differences in the experience of attentional lapses.

Across both studies, we found that controlling for attentional lapses affected the WPR more strongly on the level of covariances than on the level of correlations. This result has important theoretical implications, because it indicates that the occurrence of attentional lapses affects the inter-individual variance in the right tail of the RT distribution. In other words, inter-individual differences in attentional lapses affected the amount of between-subject variability in the right tail of the RT distribution and could thus account for a large part of the WPR on the level of covariances. On the level of correlations, however, they only accounted for a small part of the WPR, because here the WPR was calculated based on standardized measures (i.e., controlled for between-subject variability in RTs). The idea that between-subject variability may differ across RT bands is not new (see Coyle 2003; Larson and Alderton 1990). The present study demonstrates that these differences in between-subject variability across RT bands are not merely a statistical artifact, but substantially related to individual differences in elementary attentional processes.

However, there is an alternative and simpler mathematical explanation that could account for the different results on the level of covariances and correlations. We found that RTs in faster and in slower trials are highly correlated. In consequence, it is plausible that fast responses are nearly proportional to slow responses. Furthermore, the nature of slower RTs is that their variance is larger in comparison to faster responses. Consequentially, we would assume that individual differences in RTs would fan out and the variance of individual differences become larger in slower RTs. Given that the intelligence score of each individual remains the same while the RT variance increases over the RT distribution, the covariance between intelligence and RTs grows monotonically larger towards slower RTs. In contrast, correlations would not necessarily increase in the same pattern, because they are standardized. Considering this pure mathematical explanation of the different results in covariances and correlations, one could either conclude that covariances are more sensitive than correlations or that correlations are more reliable than covariances.^1^

Our results are in line with Coyle’s (2003) claim that the WPR is not driven by outlier or extreme values. Depending on the task, we extracted a certain number of trials out of the middle of participants’ RT distributions. Additionally, we applied a careful intra- and inter-individual outlier analysis. In both studies, we found a robust increase of the magnitude in covariances that is consistent with the WPR. Moreover, we found a significant WPR effect on the standardized/correlational level in Study 2. In contrast, we did not find this significant worst performance pattern in the correlations in Study 1. Possible reasons for this may be the already discussed low statistical power and small sample size. However, we clearly observed a similar course of correlations over the RT distribution in both studies (see Figure 3 and Figure 6). Notably, several previous studies used a descriptive approach for specifying the WPR. Although a test of significance is certainly warranted to test the existence of the WPR against chance (see Frischkorn et al. 2016), it is not uncommon to rely on descriptive evidence for the investigation of the WPR.

Effect sizes of the moderating role of the attentional lapses covariates on the WPR were small. Some of these estimates were η²part < 0.01, especially in the analyses with standardized coefficients, which should be interpreted as very small effects. The reason why those small effects were significant is that those interaction terms were tested with a very large number of degrees of freedom, due to the trial-by-trial analyses and the repeated-measures design. As a consequence, the standard errors became very small and small *b*-weights reached the significance level more quickly. This may be considered as curse and blessing at the same time. On the one hand, we had enough power to detect small influences of attentional lapses on the WPR; on the other hand, statistical tests may have been overpowered, leading to the adoption of irrelevant effects as an explanation for the WPR. That is, the multilevel approach to the WPR is a powerful instrument that bears the risk of overpowering. An alternative approach could be to use Fisher’s *Z*-test (e.g., Edwards 1976) as a more conservative method, which has less statistical power but requires a problematic two-stage estimation processes to assess the statistical significance of the WPR. 

However, especially in study 2 some significant parts of the worst performance pattern remained unexplained after controlling for attentional lapses. It is important to conclude that some parts of the increasing magnitude in covariances and correlations between RTs and intelligence could not be explained by attentional lapses. There could be additional reasons for the origin of the WPR.

### 4.1. Alternative Accounts of the Worst Performance Rule

Beyond the attentional lapses account, there are two prominent alternative explanations of the WPR. They cannot be rule out as alternative explanations by our findings. To some degree these accounts are additional explanations for the remaining unexplained parts of the worst performance patterns and to some other degree they complement each other and can even be transferred into each other.

The *drift diffusion model account* claims that inter-individual differences in the evidence accumulation process could explain the WPR (Ratcliff et al. 2008). The drift diffusion model is a mathematical model that describes binary decision making as a random walk process through which evidence is accumulated until one of two decision thresholds is reached (Ratcliff 1978). The basic diffusion model consists of four parameters, namely the drift rate, which describes the strength and direction of the evidence accumulation process, the boundary separation, which describes how much information needs to be accumulated before a decision is being made, the starting point, which describes biases in decision making, and the non-decision time, which encompasses the time needed for all non-decisional processes such as encoding and response execution. The drift rate parameter in particular has been repeatedly shown to be associated with individual differences in mental abilities, working memory capacity, and intelligence (Ratcliff et al. 2010, 2011; Schmiedek et al. 2007; Schubert et al. 2015). More intelligent individuals show higher drift rates across several tasks (Schmiedek et al. 2007; Schubert et al. 2015, 2016). In their simulation study, Ratcliff et al. (2008) showed that the drift rate parameter of the diffusion model is more negatively related to slower quantiles compared to faster quantiles of the RT distribution, which means that the drift rate parameter and its underlying processes were better described by slower compared to faster RTs. The drift rate parameter is typically considered as a measure of the speed of information uptake. Hence, it is possible that the speed of information uptake is more validly measured in slower responses, which in turn would lead to higher negative correlations between RT and intelligence in slower than in faster responses. The higher validity of slower responses for the speed of information uptake could be an alternative explanation of the WPR. In other words, one could say that individual differences in the speed of evidence accumulation (measured by drift rates) may also account for the pattern of the WPR, as they give rise to individual differences in slowest RTs and are also strongly related to individual differences in cognitive abilities. However, drift rates are likely affected by a number of lower-level cognitive processes that may also include attentional processes. The drift diffusion model account of the WPR is not necessarily irreconcilable with the attentional lapses account. In this sense, it is also possible that attentional lapses are related to differences in the evidence accumulation process (see also Boehm et al. 2021).

Another explanation of the WPR focuses on its statistical characteristics (Sorjonen et al. 2020, 2021). With simulated data, Sorjonen et al. showed that the WPR is a special case of the *correlation of sorted scores rule* (Sorjonen et al. 2020, 2021). This rule states that the correlation between a sorted measure of performance (e.g., binned mean RTs or trial-wise sorted RTs) and intelligence will depend on the direction of the correlation between the variability in performance (e.g., intra-individual standard deviation in RTs) and intelligence. Because of the negative correlation between intra-individual standard deviation in RTs and intelligence, the rule predicts the emergence of the WPR. If there were a positive correlation between intra-individual variability in the respective performance measure and intelligence, the rule would instead predict a best performance rule. It is well-established that more intelligent individuals show a smaller standard deviation in RTs (Doebler and Scheffler 2016), which was also the case in our sample. We found negative correlations between the variance in RTs and cognitive abilities, *r* = −.30, *p* = .003, in Study 1, and from *r* = −.20 to *r* = −.25, all *p*s < .001, in Study 2. Hence, the WPR could also be (statistically) accounted for by the correlation of sorted scores rule. In turn, the correlation of sorted scores rule does not rule out the attentional lapses account of the WPR, because it is possible that the larger intra-individual RT variability in individuals with lower cognitive abilities results as the consequence of their more frequent experience of attentional lapses.

### 4.2. The Curious Course in Very Slow RTs

A novel and surprising finding in this study was the observed decrease in the magnitude of negative correlations and the simultaneous accelerated increase in the magnitude of negative covariances, respectively, in the slowest 15 percent of the responses (Figure 3 and Figure 6). Apparently, some unknown process unrelated to intelligence increased the variance in RTs in the right tail of the RT distribution, which puts the WPR in a different light. Our observations are consistent with the meta-analysis of Schubert (2019), who described a logarithmic trend of the increases in the magnitude of negative correlations. This meta-analysis found that the increases in the magnitude of negative correlations is largest from the fastest to the mean performances and flattens from the mean to the slowest performances. Because of this observation, it was suggested to rename the WPR as *the not-best performance rule*, which is arguably a more appropriate name for this phenomenon. Welhaf et al. (2020)^2^ replicated the not-best performance rule. With our trial-by-trial analyses, it was possible to draw a more detailed picture of this phenomenon and we found Schubert’s (2019) observed logarithmic trend of correlations over the RT bins. There was an unexpected decline in the negative correlations in the slowest trials. Surprisingly, the increase in covariances accelerated at the same time. Based on these observations, we can conclude that some unknown process unrelated to cognitive abilities gave rise to RT variance in the slowest responses. The observed decline in correlations is also consistent with many previous studies that revealed a decrease or stagnation in the magnitude of the negative correlations in the slowest RT bins (Fernandez et al. 2014; Ratcliff et al. 2010; Salthouse 1998; Saville et al. 2016; Schmitz et al. 2018). Taken together, it seems that our observation is not an isolated case but a replicable phenomenon. Further studies may address the reasons for this conundrum.

## 5. Conclusions

Taken together, our results support the attentional lapses account of the WPR. Using multilevel models, we demonstrated that different single measures of attentional lapses accounted for some parts of the increasing magnitude in covariances and correlations between intelligence and RTs from the fastest to the slowest responses. The combined influence of several self-reported and objective attentional lapses measures accounted fully for this phenomenon, which in turn underlines the multiverse nature of the attentional lapses construct. Our results suggested that the WPR is caused by inter-individual differences in attentional lapses. Thus, it seems that individual differences in attentional control processes are an important factor contributing to individual differences in cognitive abilities.

## Figures and Tables

**Table 1:** Descriptive statistics of all variables.

	Mean	*SD*	Reliability	*N*
ACC	96	2	---	85
RT	836.69	154.06	.99	85
Intelligence	1498.29	80.02	.79	85
IQ	94.58	16.12	.79	85
TUT	26.07	19.24	.96	85
Q-SMW over all	37.64	8.88	.81	85
Q-SMW/item	5.38	1.29	---	85
MRT	73.49	29.45	.99	85
P1 amplitude	0.94	1.34	.96	84
P3 amplitude	3.91	2.97	.99	84
Alpha power	1.20	0.94	.92	84
Theta power	0.00	0.84	.72	84

Note: ACC = percent of correct responded trials, RT = reaction time in ms (340 trials of each subject were included), Intelligence = sum score of all scales of the Berliner Intelligence Structure Test, IQ = the intelligence sum score transformed to an IQ score, TUTs = percentage of task-unrelated-thoughts, Q-SMW = mean score in the questionnaire measuring spontaneous mind wandering, MRT = response variability in ms in the metronome response task, P1 = mean amplitude of the occipital P1 in microvolts, P3 = mean amplitude of the centro-parietal P3 in microvolts, Alpha = mean parieto-occipital alpha power in decibel before an imperative stimulus was presented, Theta = mean fronto-central theta power in decibel after an imperative stimulus was presented, reliability: either estimated with the Spearman-Brown corrected correlation coefficients based on an odd-even split (RT, TUTs, MRT, P1 amplitude, P3 amplitude) or with Cronbach’s α (Intelligence test score, Q-SMW, Alpha power). Theta power reliability was estimated by the correlation between the two corresponding electrodes.

**Table 2:** Correlations between all variables.

	1	2	3	4	5	6	7	8	9
1. Mean RT									
2. *SD* RT	.86 ***								
3. Intelligence	−.29 **	−.30 **							
4. TUT	−.12	−.27 *	.15						
5. Q-SMW	−.11	−.04	.09	.30 **					
6. MRT	.31 **	.32 **	−.27 *	−.03	−.11				
7. P1 amplitude	−.11	−.06	.03	−.02	.06	−.22 *			
8. P3 amplitude	.03	.03	−.05	.01	−.07	−.02	.27 *		
9. Alpha power	−.18	−.16	.03	−.11	−.13	.06	.06	.02	
10. Theta power	−.18	−.19	.18	.09	.09	.03	−.09	−.16	−.05

Note: Mean RT = mean reaction times (340 trials of each subject were included), *SD* RT = standard deviation of reaction times (340 trials of each subject were included), TUT = mean rate of task-unrelated thoughts, Q-SMW = mean score in the questionnaire for spontaneous mind wandering, MRT = response variability in the metronome response task, P1 amplitude = mean amplitude of occipital P1, P3 amplitude = mean amplitude of centro-parietal P3, Alpha power = mean pre-fixation cross alpha power, Theta power = mean post fixations cross theta power, * *p* < .05, ** *p* < .01, *** *p* < .001.

**Table 3:** Baseline multilevel model of the WPR on an unstandardized level.

RT On	*b-*Weight (Standard Error)	*df*	*t*-Value	Random Effect *SD*	*p*
Intercept	835.82 (15.86)	85	52.62	146.45	<.001
intelligence	−44.18 (15.98)	85	−2.77		.007
trial number	146.99 (5.20)	85	28.26	47.95	<.001
trial number × intelligence = WPR	−14.93 (5.23)	85	−2.85		.005

Note: *N* = 85. 340 trials of each participant were included for analysis. Data were centered to the trial with the sorted number of 170 and afterwards rescaled between −2 and 2. A significant interaction between *trial number* and *intelligence* represents a significant increase of the magnitude in covariation according to the WPR.

**Table 4:** Full multilevel model, which tests the effect of attentional lapses covariates (TUTs + Q-SMW + MRT) on the WPR on an unstandardized level.

RT On	*b-*Weight (Standard Error)	*df*	*t*-Value	Random Effect *SD*	*p*
intercept	835.82 (15.40)	85	54.29	96.56	<.001
intelligence	−44.18 (15.49)	85	−2.85		.005
trial number	146.99 (4.91)	85	29.91	47.38	<.001
control	−835.82 (0.27)	57630	−3091.39		<.001
trial number × intelligence = WPR	−14.93 (4.94)	85	−3.02		.003
intelligence × control	15.10 (0.27)	57630	55.53		<.001
trial number × control	−146.99 (0.23)	57630	−627.78		<.001
trial number × intelligence × control	6.05 (0.24)	57630	25.70		<.001

Note: *N* = 85. For each participant, 340 trials were included in the analysis. Data were centered to the trial with the sorted number of 170 and rescaled between −2 and 2. *Control* is a dummy coded factor, which represents raw RTs or RTs residualized by the corresponding attentional lapses covariates. A significant three-way interaction between *trial number*, *intelligence* and *control* represents a moderating influence of the covariates on the covariance.

**Table 5:** Descriptive statistics of all RT variables in Study 2.

	Mean	*SD*	Reliability	*N*
RT AF	461.03	49.65	.99	463
RT LF	532.35	85.93	.99	416
RT Stroop	508.34	49.86	.99	460
RT SART	510.62	81.94	.99	441

Note: RT AF = reaction time in the arrow-flanker task, RT LF = reaction time in the letter-flanker task, RT Stroop = reaction time in the number-stroop task, RT SART = reaction time in the SART, reliabilities were estimated with Spearman-Brown corrected odd-even split correlations.

**Table 6:** Correlations between all variables.

	1	2	3	4	5	6	7	8	9
1. Mean RT AF									
2. *SD* RT AF	.65 ***								
3. Mean RT LF	.53 ***	.42 ***							
4. *SD* RT LF	.34 ***	.40 ***	.73 ***						
5. Mean RT Stroop	.63 ***	.40 ***	.49 ***	.33 ***					
6. *SD* RT Stroop	.31 ***	.48 ***	.30 ***	.32 ***	.52 ***				
7. Mean RT SART	.11 *	−.04	.12 *	.05	.24 ***	.02			
8. *SD* RT SART	.13 **	.18 ***	.14 **	.16 **	.23 ***	.28 ***	.21 ***		
9. WMC	−.20 ***	−.22 ***	−.19 ***	−.20 ***	−.23 ***	−.25 ***	−.01	−.23 ***	
10. TUT	.12 *	.20 ***	.19 ***	.26 ***	.16 **	.22 ***	−.02	.21 ***	−.23 ***

Note: Mean RT AF = mean reaction times in the arrow-flanker task, *SD* RT AF = standard deviation of reaction times in the arrow-flanker task, Mean RT LF = mean reaction times in the letter-flanker task, *SD* RT LF = standard deviation of reaction times in the letter-flanker task, Mean RT Stroop = mean reaction times in the number-stroop task, *SD* RT Stroop = standard deviation of reaction times in the number-stroop task, Mean RT SART = mean reaction times in the SART, *SD* RT SART = standard deviation of reaction times in the SART, TUT = task unrelated thoughts, WMC = working memory capacity, * *p* < .05; ** *p* < .01, *** *p* < .001.

## Data Availability

The preprocessed data supporting the findings of Study 1 and the code for the statistical analysis used in this manuscript are available via the Open Science Framework (https://osf.io/5pafg/, accessed on 23 December 2021). Access to raw data of Study 1 will be granted upon request. The data supporting the findings of Study 2 are available via the Open Science Framework (https://osf.io/9qcmx/, accessed on 5 February 2021).

## Notes

1 Many thanks to an anonymous reviewer for this suggestion.

2 We used the same data used by Welhaf et al. (2020) in Study 2.

## Supplementary Materials

The following figure and tables are available via the Open Science Framework (https://osf.io/5pafg/, accessed on 23 December 2021), Figure S1: EEG Electrode assembly; Tables S1–S38: Results of multilevel analyses.
